# Ten simple rules for interpreting and evaluating a meta-analysis

**DOI:** 10.1371/journal.pcbi.1011461

**Published:** 2023-09-28

**Authors:** Rebecca B. Carlson, Jennifer R. Martin, Robert D. Beckett

**Affiliations:** 1 Health Sciences Library, University of North Carolina at Chapel Hill, Chapel Hill, North Carolina, United States of America; 2 Health Sciences Library, The University of Arizona, Tucson, Arizona, United States of America; 3 Department of Pharmacy Practice and Science, R. Ken Coit College of Pharmacy, The University of Arizona, Tucson, Arizona, United States of America; 4 Department of Pharmacy, Parkview Health, Fort Wayne, Indiana, United States of America; Carnegie Mellon University, UNITED STATES

## Introduction

A systematic review with meta-analysis is often considered the highest level of evidence in ranking the literature [1]. The term “systematic review” refers to the overall publication, while the term “meta-analysis” indicates that the review includes a statistical synthesis of results from at least 2 of the included studies [2]. Not all systematic reviews include a meta-analysis, but meta-analyses are always within a systematic review. There are different types of meta-analyses in which statistical analysis can be performed, including network meta-analysis and Bayesian meta-analysis [3]. While these meta-analysis methods can vary, the majority are “variations on a weighted average of the effect estimates from the different studies [2].”

Their purpose is to present structured and statistical methodologies to synthesize evidence that can be used to make informed decisions, and, ideally, they include a large sample size of studies that can be summarized to answer a research question [4]. For readers, a properly conducted meta-analysis with a structured and transparent approach can provide multiple benefits over other literature reviews, such as reduced bias, enhanced generalizability, increased statistical power, and overall providing efficient knowledge dissemination, and informing design of future research [1,5].

Meta-analyses provide important syntheses of published study data for evidence-based practice, research, and teaching. A recent summary paper is available on how to conduct and write a meta-analysis paper, [6] but guidance on reading and interpreting meta-analyses is scattered across many publications in various disciplines. This paper focuses on simple rules for scholars or trainees to refer to when determining if a meta-analysis is high quality. These 10 simple rules provides guidance on reading and interpreting meta-analyses, in order, from the introduction of the paper through the methods, results, and analyses, so readers can determine if the research they are evaluating contains high-quality and reliable evidence for research or practice.

### Rule 1: Gather and understand prerequisite context

When starting to read a meta-analysis, the importance of the topic and the review question are critical and should be defined in the beginning paragraphs of the paper, so the audience can carefully evaluate them within the context of the published paper, checking the quality of the methodology as well as the merit of the study [4]. Look for the rationale to focus on the research question, be well defined, and include all elements of the question or hypothesis, such as the population(s), intervention(s) or exposure(s), comparison group(s) if any, and the outcome(s) of interest [4,7]. It should include a clear statement of purpose, as well as the quality and scope of studies included [5], and address a sensible question and include studies that are comparable in quality [8].

When reviewing a meta-analysis, determine the quality of the study conducted, checking that the methodology was conducted with transparency and is reproducible [1,9]. Determine the quality of the reporting, [9] ensuring it follows the standard reporting structures recommended by Cochrane [2] and the PRISMA 2020 guidelines [10], for example, or by any other applicable, discipline-specific guidelines.

Author affiliations, as well as conflicts (or competing or declarations) of interest, also should be taken into consideration when reviewing a meta-analysis. Disclosure of conflicts of interest and the minimization of author biases is important to scientific rigor and credibility of a review [7]. The team of researchers conducting the meta-analysis should have included experts without any financial or intellectual bias in the scope of the project to reduce bias. Any personal interest that could influence the study and readers should look for such information should be disclosed in the article [2,7].

### Rule 2: Appraise the quality and relevance of the search

Within the methods section, determine if the literature search process for meta-analyses is a comprehensive search to find all potentially relevant studies [11]. For example, the Cochrane methodology guidelines generally recommend searching at least 3 bibliographic databases [2,11]. A fully comprehensive search encompasses searching beyond bibliographic databases (e.g., hand searching) and include unpublished studies [2] that provide statistical information to include in the overall analysis. A comprehensive search conducted by an information expert or librarian helps to minimize bias and improve the quality of the results [12,13] along with documentation on who designed and performed the search [7].

Methodology and reporting guidelines call for the documentation of the search syntax used for all databases [10]; this is important for readers to verify the comprehensiveness of the search strategies and ensure research reproducibility and transparency [13]. Check to verify that the literature search terms were included in an appendix or elsewhere in the supplemental materials of the article, included subject heading terms or controlled vocabulary, and a comprehensive list of keywords describing each concept [10]. Filters or limits in the search are recommended to be validated filters to identify a specific subset of information, such as randomized controlled trials [2]. Determine if they were validated filters and if they were cited by the authors.

### Rule 3: Evaluate methods used for citation screening and data extraction

The methods section of a systematic review and meta-analysis detail the steps to systematically include and exclude studies and can help readers assess their quality. To begin, determine if clear a priori definitions of the eligibility criteria were used and match the purpose and research question(s) of the review [8,11]. These inclusion and exclusion criteria determine which studies are included in a systematic review and eligible for the meta-analysis. Having these defined before any studies were evaluated for inclusion is important for reducing the risk of biased results. Check for documentation in the methods section of an unbiased and reproducible screening process for each study found in the literature search [4] and details on how every paper was evaluated according to the authors’ inclusion criteria [10]. Beyond a description of an independent and systematic study selection process, determine measures of inter-rater reliability, or agreement between 2 reviewers on the application of the inclusion criteria. High inter-rater reliability allows for increased confidence in the methods of data inclusion [4]. Satisfaction of these elements can help effectively interpret and apply results, as they provide insight into the study population, and appraise study quality.

A clear description of the process for data extraction should also be reviewed in the methods section. Determine if the data extraction process was completed independently by 2 reviewers [14,15]. Readers should check that this dual data extraction process was completed because having only 1 person extract data for each study, as opposed to 2 people, has been shown to lead to more errors in the results, and these mistakes in collecting data could impact the effect estimates in the overall meta-analysis [15]. The authors should also indicate any software package and version used to manage the search and conduct the analyses. In a recent cross-sectional study, the most common analysis packages were Review Manager, used by the Cochrane Collaboration, Stata, R, and Comprehensive Meta-Analysis [16]. Other software was less prevalent and may have less of a niche focus on meta-analyses.

### Rule 4: Determine the risk for inherited limitations

Meta-analyses, as retrospective syntheses of research, are at risk for inherited limitations; in other words, the quality of a meta-analysis is directly proportional to the quality of the original studies included [11]. If an original study is of poor quality or affected by methodological biases, including it in a meta-analysis will bias the meta-analysis [11] and can lead to overestimates of treatment effects [17]. This means quality assessment and risk of bias assessment is an important checkpoint in the paper’s methods section, as it can impact the validity and relevance of the results [11,14]. In the methods section, look for a clear description of study evaluation using a validated instrument, such as the Cochrane risk-of-bias tool for randomized controlled trials in clinical applications. This description might be called a quality assessment or a risk of bias assessment; while these terms are often used interchangeably, technically, quality assessment is an examination of methodological safeguards against bias in a study, and risk of bias assessment is the implication of the presence or absence of those safeguards [18]. Regardless of terminology, readers should look to see that a complete assessment of the “design, conduct, and analysis of a study” was completed for each study using a validated instrument tailored to the study design [18].

Validated tools are available across many disciplines and study types, so check that appropriate tools were applied to evaluate the relevant potential points of bias for those papers’ methodologies. Also, missing data from original studies can be problematic, especially when that data relate to the outcomes being measured in the meta-analysis; thus, investigators should address how any missing data were handled [2]. Some risk-of-bias tools address missing data as well. Finally, review the results of that quality assessment, study by study and point by point, to determine the risk for inherited limitations or biases in each study included in the review. Ultimately, for a true assessment of risk for inherited limitations, the reader may need to individually review key included studies.

### Rule 5: Assess potential for publication bias

Publication bias is a significant threat to the validity of a meta-analysis [19]. This bias can be understood as a systematic error in a meta-analysis that occurs because not all the evidence is properly represented. Although there can be both over- and underrepresentation of evidence, underrepresentation is more prominent and occurs when researchers do not submit their study results to a journal or submit only part of their results, journals decide not to publish negative results or other studies, or the study retrieval and selection procedures of a meta-analysis do not include a publication [11,19]. When any of these practices occur, the results of a meta-analysis may not be representative of the totality of the literature [12] and may be overestimating the true effect of the intervention [20]. For example, in medical meta-analyses, those who rely on their results to make point-of-care decisions could potentially cause harm to patients when publication bias occurs [21]. Across all disciplines, biased results of a meta-analysis may cause readers to draw mistaken conclusions about the totality of the literature. Check for publication bias, and look for proof in the methods section that the authors completed a comprehensive literature search, including looking beyond published journal articles and outside of bibliometric databases to find unpublished, negative, international, or other commonly underrepresented studies [12,13].

**Fig 1:**
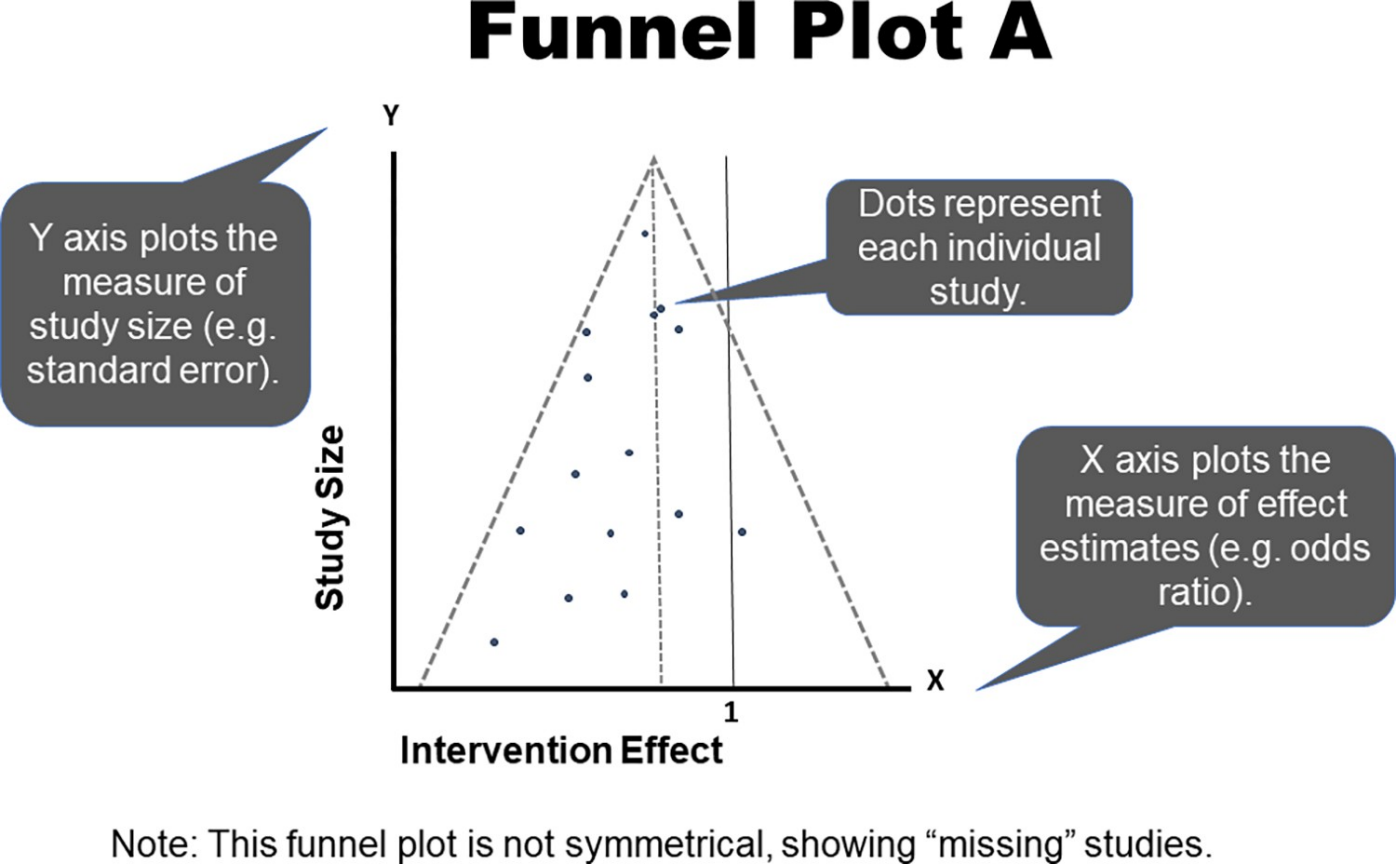
Funnel Plot A.

**Fig 2:**
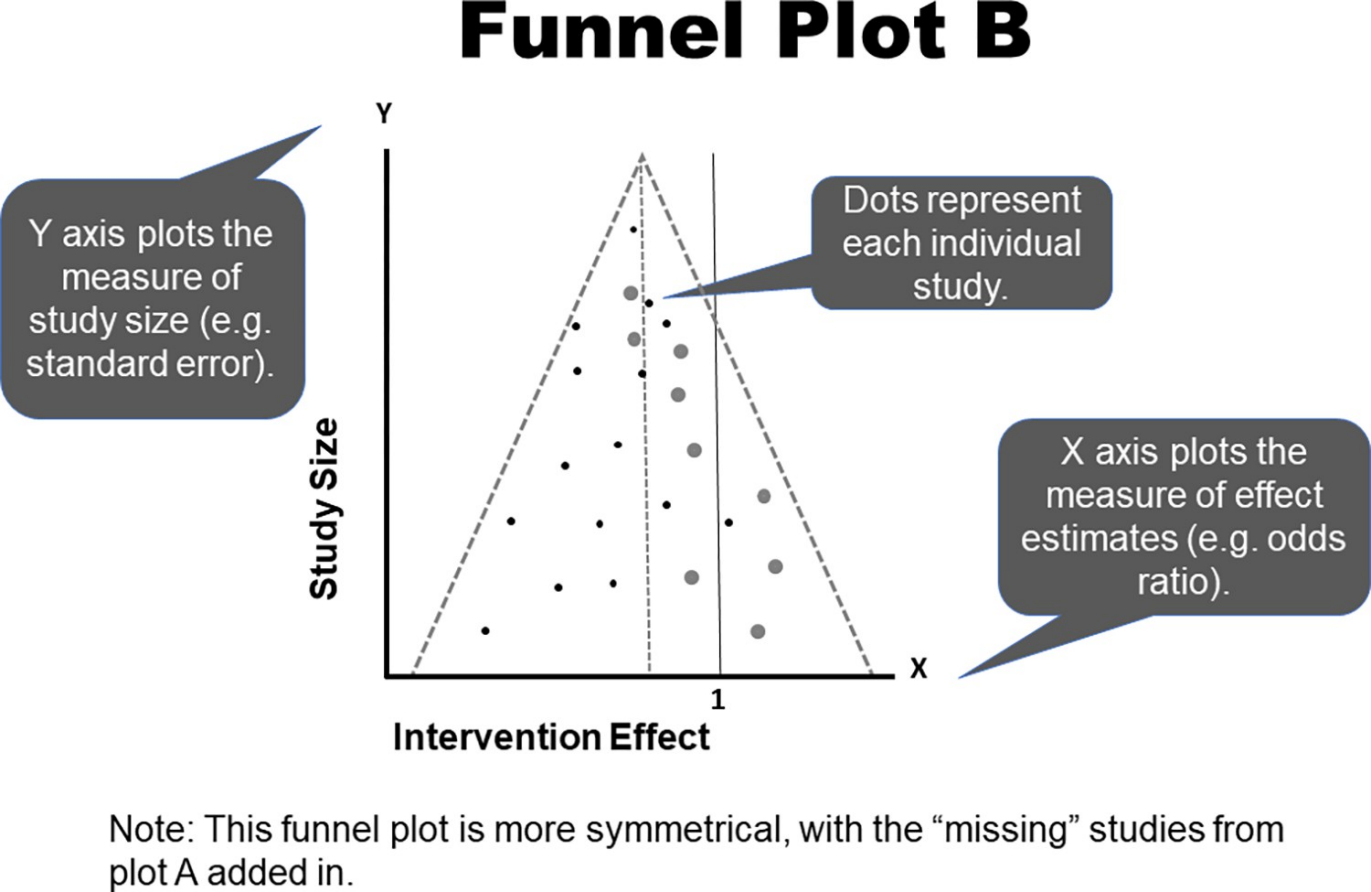
Funnel Plot B.

Presence of publication bias can also be evaluated by readers through review of a funnel plot. This figure plots a measure of observed effect size (usually log transformed) from each included study on the x-axis against a measure of sample size (e.g., within-study variance) on the y-axis [19]. Theoretically, the largest study should have the observed effect size closest to the synthesized “true” effect, and then the smaller studies should be symmetrically distributed relative to that summary estimate, forming a triangular, “upside down funnel” shape [14,19]. However, if publication bias has impacted the study, the plot will not form this shape because of “missing” studies from one side or the other. These gaps would suggest that certain evidence is over- or underrepresented in the study, and, generally, readers should be cautious that the results may not represent the most conservative estimate of the overall effect. Two examples of funnel plots are shown in Figs 1 and 2.

Interpretation of funnel plots may be subjective. Therefore, readers should interpret results with caution, especially if there are fewer than 10 studies in the sample [14,22]. This is underscored by results from a validation study that found funnel plots to be associated with the least reproducible estimates of publication bias by users [19]. Though commonly cited as important aspects of meta-analyses, funnel plots are infrequently presented. An Egger test may also be provided, with statistically significant results suggesting asymmetry and, therefore, publication bias are present; however, readers should focus on visual interpretation of the plot because of risk that the Egger test may not be sufficiently powered [14]. See Table 1 for a definition of Egger’s test statistic, and other statistical measures that appear throughout the Rules.

**Table 1:** Key statistics used in meta-analyses [8,21,23].

Statistic	Definition	Use
Absolute difference (AD) or absolute risk reduction (ARR)	Event rate difference (experimental group minus control group), pooled among studies	Illustration of absolute group differences for positive outcomes (AD) or negative outcomes (ARR)
Cochrane’s Q test statistic	*P* value (probability) test statistic indicating whether there are differences among study results unlikely due to chance alone	Heterogeneity analysis to determine validity of pooling study results; a statistically significant results indicates differences among studies
Egger’s test statistic	*P* value (probability) test statistic indicating whether there is asymmetry of study results on a funnel plot	Assessment of publication bias; a statistically significant result indicates asymmetry, which suggests publication bias
Hazard ratio (HR)	Relative risk for the outcome with the experimental group compared to the control, accounting for the factor of time, pooled among studies	Illustration of outcomes in prospective studies
I^2^	Degree of heterogeneity, or difference, among the results from studies included in a meta-analysis	Heterogeneity analysis to determine validity of pooling study results; values closer to 100% suggest more heterogeneity
Odds ratio (OR)	Relative likelihood of exposure for subjects who experienced an outcome compared to those who did not, pooled among studies	Illustration of outcomes in retrospective studies, especially case–control
Relative risk (RR) or rate ratio (RR)	Relative likelihood of the outcome with the experimental group compared to the control, pooled among studies	Illustration of outcomes in prospective studies, especially adverse outcomes
Standardized mean difference (SMD)	Weighted mean difference (see below) divided by standard deviation, pooled among studies	Illustration of results involving continuous or ordinal data that had different units of measurement
Weighted mean difference (WMD)	Pooled difference between groups (experimental group minus control group) in any measurement, pooled among studies	Illustration of results involving continuous or ordinal data that had similar units of measurement

### Rule 6: Determine whether there is methodological or subject heterogeneity

Along with publication bias and inclusion of low-quality studies, heterogeneity poses the most serious challenge to internal validity of a meta-analysis [19,20], and determining if or how much it is present in a meta-analysis is crucial for interpreting the paper’s quality. Studies included in a meta-analysis will not be identical in terms of research methods and sampled subject population. The degree of this diversity among studies is termed “heterogeneity” [1,5,19,23]; more specifically, methodological heterogeneity refers to diversity in research methods, and clinical heterogeneity refers to diversity in the studied patient populations [11,24]. Methodological and subject heterogeneity can lead to measurable statistical heterogeneity, as described in further detail in Rule 7 [24]. Alternately, the impact of methodological and subject heterogeneity can be subtle and may not manifest in detectable statistical heterogeneity (again, see Rule 7) [24]. Thus, it is important for readers to review not only the meta-analysis results but also the methods, to detect potential sources of heterogeneity.

Because the methodological and subject heterogeneity are more qualitative than quantitative, a close evaluation of the meta-analysis inclusion and exclusion criteria and overview of included studies is the best way for readers to identify potential sources of heterogeneity [8,24,25]. Significant differences among included studies in terms of how they were conducted, and their patient populations may signal that it is inappropriate for study results to be pooled and that any results should be interpreted with great caution [24]. Examples of potential sources of methodological heterogeneity include differences in study interventions, exposures, outcomes, or descriptive statistics used to illustrate outcomes [11,24,25]. Extra care is needed when studies of very diverse interventions (e.g., different medications within the same therapeutic class) that seek to achieve the same outcome are pooled, so confirm that these study attributes are not significantly different [25].

Examples of potential sources of subject heterogeneity include differences in intervention target definitions, disease state or exposure severity, inclusion and exclusion criteria, and baseline characteristics [11,25]. While some of these examples come from medical meta-analyses, the same principles can be applied outside of health disciplines to meta-analyses that answer nonclinical questions; no matter the discipline, readers should examine whether subject criteria and characteristics are consistent. When readers go to apply the results in research or practice settings, it is important to closely review information provided about the individual study populations to appraise for situationally relevant differences between the reader’s population of interest and the studied population(s) [11]. Overall, the critical reader should qualitatively consider whether it was appropriate to pool studies and whether there is too much potential heterogeneity, whether or not the differences among studies led to detectable statistical heterogeneity (see Rule 7) [25].

### Rule 7: Determine whether there is statistical heterogeneity

Statistical heterogeneity can be thought of as variation among results (e.g., whether there is a statistically significant difference between groups, degree of observed effect) from the included studies and ideally should be no more than what would be expected based on chance alone [5,25,26]. If significant statistical heterogeneity is detected, the reader should apply results in practice only with extreme caution. Statistical heterogeneity can be visually examined in a meta-analysis Forest plot by drawing a dotted line through the center of the summary diamond (often done by the investigators but can be done by readers too) and analyzing whether the 95% confidence interval bars from each individual study cross the dotted line and overlap with each other. If they do, heterogeneity is expected to be low [19,25,26]. Forest plots have been demonstrated as the most reproducible visual illustration of heterogeneity and should be examined carefully by readers [19].

Statistical heterogeneity should have been statistically examined by the investigators using the chi-squared test (also referred to as Cochrane or Cochran Q test, chi-squared test for heterogeneity, or chi-squared test for homogeneity) or the I^2^ or Higgins I^2^ statistic [23,25,26]. These assessments indicate whether this type of heterogeneity is present and to what degree, respectively [14]. *P* values from a chi-squared test for heterogeneity can be interpreted similarly to standard rules, though a higher than usual alpha value (i.e., 0.1 instead of 0.05) may be used because the test may be insensitive to significant findings. A high *p*-value suggests low risk for heterogeneity, while a low *p*-value suggests higher risk [24,25]. The I^2^ result is provided as a percentage ranging from 0% (all variation is expected to be due to chance) to 100% (no variation is expected to be due to chance) [5,26]. It should be noted that statistical testing for heterogeneity is not recommended when the meta-analysis contains fewer than 5 studies, because of the risk for erroneous results [22]. Therefore, the reader should always be mindful of the number of included studies before drawing firm conclusions about statistical heterogeneity.

There is no consensus on what I^2^ value constitutes a threshold for concern [24]. An I^2^ result greater than 50% is considered cause for concern that the degree of heterogeneity may not be acceptable, while “moderate” values fall between 50% and 75% [14,26]. Values in the range of 30% to 60% have also been deemed “modest” [20], while values less than 15% to 25% may be considered “low,” and greater than 75% to 80% “high” [14,24,25].

Statistical methods commonly used in meta-analyses are defined in Table 2. If substantial heterogeneity is not present, a fixed effects model may have been used by the investigators to synthesize study results. This model assumes that chance is the only important source of variation among studies [14,23,25,26]. However, if substantial heterogeneity is present, it is important for readers to verify in the methods that investigators used a random effects model to carry out the meta-analysis. This model is more conservative and accounts for the possibility that factors besides chance, such as differences in study methods and patient populations, contributed to variation among studies [14,19,23,25,26]. It may also have been appropriate for the investigators to not pool results at all when heterogeneity is high [25]. If substantial heterogeneity is present, the reader should examine whether investigators attempted to explore potential sources of heterogeneity using meta-regression and/or subgroup analysis (see Rule 10) [1,11,14,19,20,24].

**Table 2:** Key statistical methods used in meta-analyses [21].

Method	Definition	Use
Fixed effects modeling (e.g., inverse probability method, Mantel–Haenszel method, Peto method)	Pools results from multiple studies, assuming all are measuring the same treatment effect	Appropriate when there is low heterogeneity and high likelihood that all relevant studies were included
Random effects modeling (e.g., DerSimonian and Laird method)	Pools results from multiple studies, assuming they represent distribution of results among a range of values	Appropriate when there is moderate heterogeneity and low likelihood that all relevant studies were included
Individual patient data meta-analysis	Pools results for individual patients rather than group results	Allows for investigation of more precise subgroup and group differences but may be more subject to differences in individual study methodology
Meta-regression	Pools results while accounting for specified study characteristics when estimating effect size	Account for potential differences among studies (e.g., randomization, control)

Regardless of whether statistically significant heterogeneity is detected, and especially if it is more than “low,” it is good practice to closely review the included studies for potential sources of subject or methodological heterogeneity (see Rule 6) [11,20,25]. They may not always be detected using statistical heterogeneity assessments, so readers must assess the meta-analysis with their own application needs in mind [24].

### Rule 8: Appraise the statistical significance of the results

**Fig 3:**
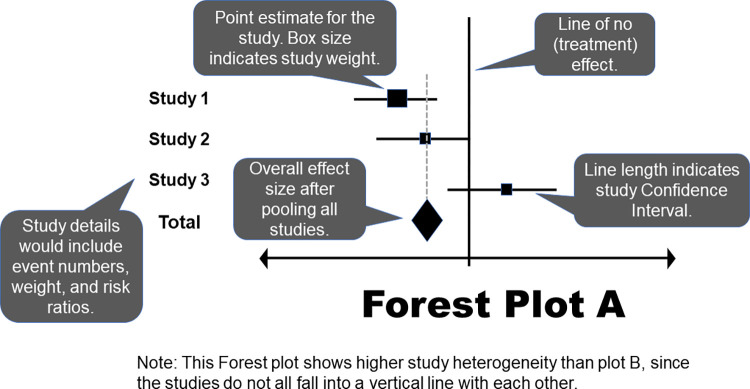
Forest Plot A.

**Fig 4:**
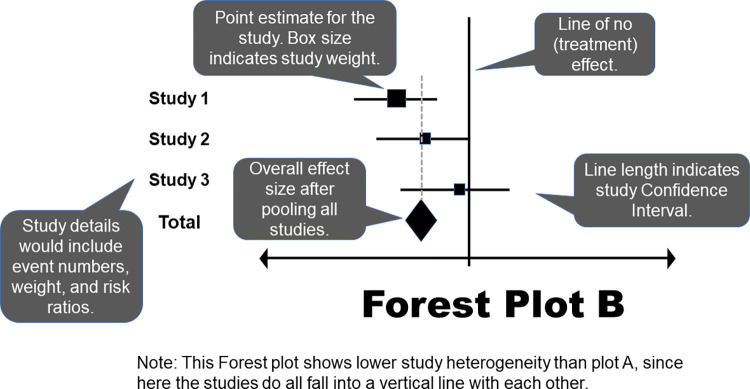
Forest Plot B.

The Forest plot, also known as the “blobbogram,” is the most common tool used to illustrate pooled meta-analysis results and aids readers in determining the statistical (i.e., are differences between groups likely due to chance alone?) and subject (i.e., if statistically significant, how substantial of an effect is there?) significance of results [5,19,24,27]. There are other figures, such as the L’Abbé plot, which can be used to illustrate meta-analysis results, though they are much less commonly employed than the Forest plot [19]. In a validation study, the highest reproducibility of heterogeneity assessment among evaluators was seen with the Forest plot, followed by the standardized residual histogram [19]. The weighted box plot had the least reproducibility. These results reinforce the value of continued use of Forest plots, and readers should use the Forest plot(s) in their determination of significance. Two examples of Forest plots are shown in Figs 3 and 4.

Typically, the first column of a Forest plot lists each study that provided results for a specific outcome or measurement, which should be clearly labeled [19,22,24,27]. The results are then graphically illustrated using dots or squares plotted against a horizontal line (one for each study), with a vertical line illustrating the point of no difference. The size of the dot or square is proportional to the sample size enrolled in the study. Sometimes, a percentage weight is provided to indicate the proportion of the sample in the overall meta-analysis that originated from each study. This can be used by readers to assess whether it is likely that 1 or 2 studies were likely responsible for the overall finding, or whether the sample of studies contributed similarly. Results may be presented using a variety of summary statistics, but most common are relative risks, odds ratios, and mean differences (see Table 1). The 95% confidence intervals are indicated using whisker bars; if either side ends in an arrow, that indicates the width of the interval surpasses the plotted range [14,19,22,24,27]. At the bottom of the figure, there is a diamond representing the pooled study results, also known as the summary estimate, with its lateral points indicating the width of the 95% confidence interval.

Thus, readers can determine the statistical significance of individual studies and the synthesized meta-analysis results by visually comparing the 95% confidence intervals to the vertical reference line [19,22,24,27]. If the interval does not cross or contain the reference line, the difference is statistically significant. The figure is generally labeled to indicate whether the intervention or control is better performing on each side of the reference line, though caution should be used by readers when relying on these labels as they are often misplaced. The placement of the individual study point estimates and confidence intervals can also provide a quick visualization of statistical heterogeneity [19]. If the individual study results fall in a roughly vertical line with each other and overlap, that suggests low heterogeneity. In addition to the visualization, numerical results are provided, generally in a right-hand column. Point estimates and 95% confidence intervals for individual studies and the pooled study results also allow the reader to quickly determine whether differences are statistically significant [19,22,24,27]. Statistical tests and resulting test values should be indicated for the overall effect and for heterogeneity, usually as a footnote [24].

It has been recommended that meta-analysis researchers avoid ordering studies in a Forest plot in an arbitrary manner, such as alphabetically; rather, they should order results by date of publication or effect size to show spot trends and differences among studies’ results [22]. It is also good practice to divide Forest plots based on subtypes of studies. For example, in healthcare applications, whether results from observational studies differed from clinical trials. Additionally, Forest plots with summary diamonds should not have been prepared when only a single study (or potentially fewer than 3) has evaluated a specific endpoint as these plots can mislead the reader into concluding that these were new findings from the meta-analysis. Examine for whether plots adhere to these principles as part of their evaluation, especially for studies not published as part of the Cochrane collaboration. In an evaluation study, it was found that the most commonly missing elements from Forest plots included the number of participants in each study and the overall meta-analysis, quantitative study results, and the statistical test used to assess the overall effect [22].

### Rule 9: Appraise the magnitude of the effect

Assessment of the magnitude of the effect by readers can be more challenging when reviewing a meta-analysis compared to the original study, partially because it is possible that most or even all included studies may not have detected a difference between groups on their own [14]. The meta-analysis will have increased power though, making detection of smaller differences more likely [24]. It is critical to not just assess statistical significance of results but to carefully evaluate the magnitude of the effect to determine how meaningful it would be outside the confines of a carefully controlled study. It is also important to assess whether results from categorical data are being presented as absolute or relative numbers. If only relative numbers are provided, the reader should consider the results in context of the proportion of enrolled subjects who experienced the outcome. For example, a relative risk of 0.75 would be substantial (for most outcomes) if 50% of subjects experienced the outcome in the control group, suggesting only 37.5% experienced the outcome in the intervention group. This is an absolute risk reduction of 12.5%. However, if only 5% of control subjects experienced the outcome, a relative risk of 0.75 suggests a reduction in risk to only 3.75%, an absolute risk reduction of 1.25%. Depending on the outcome, this result may be much less significant in practice.

When determining the practical significance of the results, a close evaluation of the meta-analysis methods is also essential. This includes considering how well the objective and inclusion criteria align, the methods used to locate and select included studies, the potential that there are additional ongoing studies in the topic, the quality of the included articles, and the methods used to pool study results [11]. The number of included studies and potential sources of variation and partial reporting that might compromise validity should also be considered [5].

### Rule 10: Check for results from sensitivity and subgroup analyses

Compared to a randomized, controlled trial, the importance of closely reviewing sensitivity and subgroup analyses is elevated with meta-analyses. Sensitivity analyses help readers to determine how consistent the results are when baseline assumptions are altered. The larger power of meta-analyses compared to individual studies can help support stronger subgroup analyses to apply results more specifically in practice [1,8].

Meta-analysis investigators should have investigated potential sources of heterogeneity, if it is detected, by comparing study methods and settings. For example, studies may have varied by nation, design, intervention, or subject population [11,24]. Look for provision of sensitivity results where these variations are explored to help determine how best to apply results from the meta-analysis. There could also be an outlier study, such as one with higher rated risk for bias, those that are much smaller or larger in enrollment than the others, or one with more favorable results, which impacts the entire analysis. When such sources of heterogeneity are detected, readers could check to see if the authors conducted a sensitivity analysis where outliers were removed, or subgroups investigated to help improve applicability of results and inform design of future research. This involves repeating the study with removal of one or more studies to determine whether results are similar and determine if heterogeneity is reduced [11,20]. These subgroup analyses may also be planned based on anticipated sources of heterogeneity [11]. However, anecdotally, results from sensitivity and subgroup analyses often are not presented in meta-analysis abstracts; thus, it is critical for the reader to review the full results to determine how best to apply results in practice.

## Conclusions

While meta-analyses are meant to be at the top of the evidence pyramid, they often fall short and do not always possess high-quality methods. Past studies of systematic reviews and meta-analyses’ methods have shown that errors in search strategies, suboptimal methods, and lack of compliance with reporting guidelines are common across fields and topics [28]. This is due in part to a lack of compliance with systematic review standards by authors and journals. A recent study found only 4 out of 10 of highly ranked journals publishing meta-analyses required adherence to the standard reporting guidelines in their fields [29]. This means it is crucial for readers to critically assess all elements of meta-analyses to determine their quality before relying on them for research or practice and that meta-analyses are only as good as their methods and included studies [30]. Following these 10 simple rules guides readers through a critical examination of a meta-analysis paper from introduction to methods to results to enable readers to more confidently determine their usefulness. A summary of the rules is available as Table 3.

**Table 3:** Ten steps to interpreting and evaluating a meta-analysis.

1. Gather and understand prerequisite context Focus on a well-defined research question and have a clear statement of purpose.
2. Appraise the quality of the search The search should be comprehensive, and details included in a supplement section of the article.
3. Evaluate methods used for citation screening and data extraction Have an unbiased, reproducible screening and data extraction process and steps to include and exclude studies with clear a priori definitions of eligibility criteria.
4. Determine the risk for inherited limitations Review results for inherited limitations or biases of included studies with a description of the evaluation using a validated instrument.
5. Assess the potential for publication bias Determine if the search included unpublished studies. The funnel plot can reveal potential publication bias while, statistically, it can be determined through an Egger’s test.
6. Determine if there is methodological or subject heterogeneity Reviewing methods and results to detect potential sources of heterogeneity as well as reviewing the inclusion/exclusion criteria and included studies.
7. Determine if there is statistical heterogeneity Forest plots can be used to visually examine statistical heterogeneity; also assess I^2^ values.
8. Appraise statistical significance of the results Forest plots will aid readers in determining the significance of the results.
9. Appraise the magnitude of the effect Evaluate the size of the effect to determine how meaningful the results would be in practice.
10. Check for results from sensitivity and subgroup analysis Review sensitivity and subgroup analysis to determine how consistent the results are when baseline assumptions are altered.

## References

[1] Mudge DW, Webster AC, Johnson DW. Pro: Meta-analysis: the case for. Nephrol Dial Transplant. 2016;31(6):875–880. doi: 10.1093/ndt/gfw091 27217392

[2] Higgins JPT, Thomas J, Chandler J, Cumpston M, Li T, Page MJ, et al., editors. Cochrane Handbook for Systematic Reviews of Interventions version 6.3 (updated August 2022). Cochrane. 2022. Available from www.training.cochrane.org/handbook

[3] Sutton A, Clowes M, Preston L, Booth A. Meeting the review family: exploring review types and associated information retrieval requirements. Health Info Libr J. 2019;36(3):202–222. doi: 10.1111/hir.12276 31541534

[4] Murad MH, Montori VM, Ioannidis JPA, Jaeschke R, Devereaux PJ, Prasad K, et al. How to read a systematic review and meta-analysis and apply the results to patient care: users’ guides to the medical literature. JAMA. 2014;312(2):171–179. doi: 10.1001/jama.2014.5559 25005654

[5] Lortie CJ, Stewart G, Rothstein H, Lau J. How to critically read ecological meta-analyses. Res Synth Methods. 2015;6(2):124–133. doi: 10.1002/jrsm.1109 26099480

[6] Forero DA, Lopez-Leon S, González-Giraldo Y, Bagos PG. Ten simple rules for carrying out and writing meta-analyses. PLoS Comput Biol. 2019;15(5):e1006922. doi: 10.1371/journal.pcbi.1006922 PubMed Central PMCID: PMC6521986 31095553 PMC6521986

[7] Institute of Medicine. Finding What Works in Health Care: Standards for Systematic Reviews. Washington, DC: The National Academies Press; 2011. 10.17226/13059 24983062

[8] Interpreting Ried K. and understanding meta-analysis graphs—a practical guide. Aust Fam Physician. 2006;35(8):635–638. 16894442

[9] Berlin JA, Rubenfeld GD, O’Cearbhaill RE, Shah AS, Fihn SD. Keeping Meta-analyses Fresh. JAMA Netw Open. 2022;5(8):e2228541. doi: 10.1001/jamanetworkopen.2022.28541 36001322

[10] Page MJ, McKenzie JE, Bossuyt PM, Boutron I, Hoffmann TC, Mulrow CD, et al. statement: an updated guideline for reporting systematic reviews. BMJ. 2020;2021 (372):n71. doi: 10.1136/bmj.n71 PubMed Central PMCID: PMC8005924 PMC8005924 33782057

[11] Yuan Y, Hunt RH. Systematic reviews: the good, the bad, and the ugly. Am J Gastroenterol. 2009;104(5):1086–1092. doi: 10.1038/ajg.2009.118 19417748

[12] Page MJ, Sterne JAC, Higgins JPT, Egger M. Investigating and dealing with publication bias and other reporting biases in meta-analyses of health research: A review. Res Synth Methods. 2021;12(2):248–259. doi: 10.1002/jrsm.1468 33166064

[13] Moreau D, Gamble B. Conducting a meta-analysis in the age of open science: Tools, tips, and practical recommendations. Psychol Methods. 2022;27(3):426–432. doi: 10.1037/met0000351 32914999

[14] Andrade C. Understanding the Basics of Meta-Analysis and How to Read a Forest Plot: As Simple as It Gets. J Clin Psychiatry. 2020;81(5). doi: 10.4088/JCP.20f13698 33027562

[15] Buscemi N, Hartling L, Vandermeer B, Tjosvold L, Klassen TP. Single data extraction generated more errors than double data extraction in systematic reviews. J Clin Epidemiol. 2006;59(7):697–703. doi: 10.1016/j.jclinepi.2005.11.010 16765272

[16] Nguyen PY, Kanukula R, McKenzie JE, Alqaidoom Z, Brennan SE, Haddaway NR, et al. Changing patterns in reporting and sharing of review data in systematic reviews with meta-analysis of the effects of interventions: cross sectional meta-research study. BMJ. 2022;379:e072428. doi: 10.1136/bmj-2022-072428 PubMed Central PMCID: PMC9679891 36414269 PMC9679891

[17] Moher D, Pham B, Jones A, Cook DJ, Jadad AR, Moher M, et al. Does quality of reports of randomised trials affect estimates of intervention efficacy reported in meta-analyses? Lancet. 1998;352(9128):609–613. doi: 10.1016/S0140-6736(98)01085-X 9746022

[18] Furuya-Kanamori L, Xu C, Hasan SS, Doi SA. Quality versus Risk-of-Bias assessment in clinical research. J Clin Epidemiol. 2021;129:172–175. doi: 10.1016/j.jclinepi.2020.09.044 . 33422267

[19] Bax L, Ikeda N, Fukui N, Yaju Y, Tsuruta H, Moons KGM. More than numbers: the power of graphs in meta-analysis. Am J Epidemiol. 2009;169(2):249–255. doi: 10.1093/aje/kwn340 19064649

[20] Esterhuizen TM, Thabane L. Con: Meta-analysis: some key limitations and potential solutions. Nephrol Dial Transplant. 2016;31(6):882–885. doi: 10.1093/ndt/gfw092 27217394

[21] Dickersin K. Publication Bias: Recognizing the Problem, Understanding Its Origins and Scope, and Preventing Harm. In: Rothstein H, Sutton AJ, Borenstein M, editors. Publication Bias in Meta-Analysis: Prevention, Assessment and Adjustments. 1st ed. Chichester, England: Wiley; 2005. p. 11–33.

[22] Schriger DL, Altman DG, Vetter JA, Heafner T, Moher D. Forest plots in reports of systematic reviews: a cross-sectional study reviewing current practice. Int J Epidemiol. 2010;39(2):421–429. doi: 10.1093/ije/dyp370 20093305

[23] Israel H, Richter RR. A guide to understanding meta-analysis. J Orthop Sports Phys Ther. 2011;41(7):496–504. doi: 10.2519/jospt.2011.3333 21725192

[24] Dettori JR, Norvell DC, Chapman JR. Seeing the Forest by Looking at the Trees: How to Interpret a Meta-Analysis Forest Plot. Global Spine J. 2021;11(4):614–616. doi: 10.1177/21925682211003889 PubMed Central PMCID: PMC8119923 33939533 PMC8119923

[25] Fletcher J. What is heterogeneity and is it important? BMJ. 2007;334(7584):94–96. doi: 10.1136/bmj.39057.406644.68 PubMed Central PMCID: PMC1767262 17218716 PMC1767262

[26] Hyde CJ, Stanworth SJ, Murphy MF. Can you see the wood for the trees? Making sense of forest plots in systematic reviews 2. Analysis of the combined results from the included studies. Transfusion. 2008;48(4):580–583. doi: 10.1111/j.1537-2995.2007.01582.x 18194387

[27] Hyde CJ, Stanworth SJ, Murphy MF. Can you see the wood for the trees? Making sense of forest plots in systematic reviews. Transfusion. 2008;48(2):218–220. doi: 10.1111/j.1537-2995.2007.01514.x 18005330

[28] Ioannidis JPA. The Mass Production of Redundant, Misleading, and Conflicted Systematic Reviews and Meta-analyses. Milbank Q. 2016;94(3):485–514. doi: 10.1111/1468-0009.12210 PubMed Central PMCID: PMC5020151 27620683 PMC5020151

[29] Hameed I, Demetres M, Tam DY, Rahouma M, Khan FM, Wright DN, et al. An assessment of the quality of current clinical meta-analyses. BMC Med Res Methodol. 2020;20:105. doi: 10.1186/s12874-020-00999-9 PubMed Central PMCID: PMC7204021 32380945 PMC7204021

[30] Murad MH, Asi N, Alsawas M, Alahdab F. New evidence pyramid. Evid Based Med. 2016;21(4):125–127. doi: 10.1136/ebmed-2016-110401 PubMed Central PMCID: PMC4975798 27339128 PMC4975798

